# Allergens Responsible for Contact Allergy in Children From 2010 to 2024: A Systematic Review and Meta‐Analysis

**DOI:** 10.1111/cod.14753

**Published:** 2025-01-19

**Authors:** Daniel Isufi, Mikkel Bak Jensen, Christoffer Kursawe Larsen, Farzad Alinaghi, Jakob Ferløv Baselius Schwensen, Jeanne D. Johansen

**Affiliations:** ^1^ Department of Dermatology and Allergy Herlev and Gentofte—Copenhagen University Hospital Copenhagen Denmark; ^2^ National Allergy Research Centre, Department of Dermatology and Allergy Herlev and Gentofte Hospital Copenhagen Denmark; ^3^ Institute of Clinical Medicine, Faculty of Health Science University of Copenhagen Copenhagen Denmark

**Keywords:** allergic contact dermatitis, atopic dermatitis, children, contact allergy, fragrance, meta‐analysis, metals, patch test, preservatives, systematic review

## Abstract

Contact allergy (CA) is a frequent condition in children; however, newer estimates of the prevalence of CA in children are lacking. Herein, we aim to provide an estimate of the prevalence of CA in children from 2010 to 2024. Two authors independently searched PubMed, Embase and Web of Science for studies reporting the prevalence of positive patch tests (PPTs) to allergens in populations including ≥ 100 children (< 18 years). Proportion meta‐analyses were conducted to calculate the pooled prevalence estimates of CA in children. Seventeen studies comprising 11 593 children undergoing patch testing with 4176 (36%) PPTs were eligible for inclusion. The allergen with the highest prevalence was nickel (11.9% [95% confidence interval [CI], 8.6%–15.71%]), followed by cobalt (6.6% [95% CI, 4.2%–9.5%]), cocamidopropyl betaine (5.5% [95% CI, 3.1–8.7]), bacitracin (5.2% [95% CI, 1.2%–10.0%]), fragrance mix I (4.8% [95% CI, 2.9%–7.1%]) and methylisothiazolinone (4.3% [95% CI, 2.2%–7.2%]). Children with atopic dermatitis had higher rates of PPTs for cocamidopropyl betaine, propylene glycol, lanolin alcohol and carba mix. Across geographical areas, higher prevalences of several allergens were found in the United States compared to Europe, for example, for methylisothiazolinone. This meta‐analysis confirmed that CA is frequent in children across geographical areas; however, differences in the frequency of top allergens depend on regulatory interventions, indicating their value.

## Graphical abstract

Meta‐analysis identifies key allergens responsible for contact allergy in children, highlighting common allergens and guiding a baseline series in children and prevention strategies.

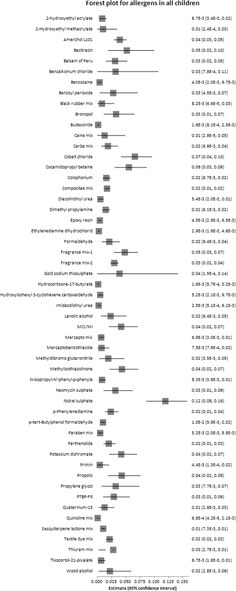

**Abbreviations**
ACD: Allergic contact dermatitis
AD: Atopic dermatitis
CA: Contact allergy
Chromium: Potassium dichromate
Cobalt: Cobalt Chloride
FM I: Fragrance mix I
FM II: Fragrance mix II
Nickel: Nickel sulphate
PPT: Positive patch test

## 1 Introduction

Contact allergy (CA) in children has for decades been accompanied by an inherent ‘uncertainty’, but it may be a more prevalent condition in children than previously anticipated. Over the past decade, significant progress has been made in understanding the allergens responsible for CA in children. Notably, CA in children is often triggered by exposure to common allergens found in cosmetics (e.g., fragrances), jewellery (e.g., nickel sulphate [nickel]) and toys (e.g., preservatives such as methylisothiazolinone [MI]) [1]. Patch testing is the gold standard for diagnosis of CA, also termed skin sensitisation. It is the first step in the diagnosis of allergic contact dermatitis (ACD), a clinical diagnosis which requires a positive patch test (PPT) to an allergen and a current or previous exposure to the allergen in question, which may partly or fully explain the dermatitis [2].

The diagnosis of CA is confirmed by a PPT reaction to the ascertained allergen. However, a uniform, standardised patch test panel for children does not exist, as is the case for adults with slight regional differences. Patch testing in children may vary significantly by region due to tradition [3, 4]. While a universally adopted European paediatric baseline series is yet to be established, a baseline series for children is under development by the European Society of Contact Dermatitis [3, 4].

A systematic review and meta‐analysis published in 2011 by Bonitsis et al. collected information about allergens responsible for CA in children in studies published until 2010 [5]. There is a need for an update, which could be considered in the standardisation of patch test panels for children in different regions. Also, political focus and regulations of several allergens may change the epidemiology of CA and ACD in children [6, 7]. Thus, in this study we aimed to provide updated evidence on the allergens responsible for CA in children between 2010 and 2024. Furthermore, we intended to evaluate the distinctions between the allergens responsible for CA in all children compared to children with atopic dermatitis (AD) only.

## 2 Methods

A study protocol was registered on the International Prospective Register of Systematic Reviews (PROSPERO; CRD42024537950). The study was conducted according to the Preferred Items for Systematic Review and Meta‐Analysis (PRISMA) guidelines [8]. A comprehensive literature search was conducted in PubMed, Embase and Web of Science for studies conducted between January 2010 and May 2024 using the search terms *‘allergic contact dermatitis’*. The articles were downloaded and uploaded to the online screening tool Rayyan [9], and duplicates were manually removed. The non‐duplicate articles were independently screened based on title and abstract by two authors (DI and MBJ), and the full text of included articles was retrieved and assessed for eligibility. Disagreement was resolved through debate with the senior author (FA).

### 2.1 Inclusion and Exclusion Criteria

To be eligible for inclusion, studies had to (I) be original, (II) written in English, (III) include ≥ 100 patch‐tested children (< 18 years), (IV) report the number of PPTs for the respective allergens and (V) include data from 2010 and onwards. If studies included data from before 2010 and after 2010, the data from 2010 onwards needed to be accessible to be eligible for inclusion. For studies reporting data in interval ranges (e.g., 2005–2012), only the data from 2010 onwards were extracted for analysis if possible. In cases where interval data were aggregated, we used the data specifically reported for the post‐2010 period if this was possible.

Exclusion criteria were: (I) non‐English articles, (II) grey literature (i.e., letters and conference abstracts) and (III) articles including < 100 children. The most comprehensive report was included in case of a population being included in more than one article.

### 2.2 Data Extraction

The following data were extracted from the included articles where possible: Author surname and year of publication, country, name of allergens, the number of patients, total number of PPTs, age and sex. Furthermore, the number of patients with AD and the number of PPTs in these patients were retrieved.

### 2.3 Statistical Analysis

Statistical analyses were performed using StatsDirect version 3.1.4 (StatsDirect Ltd., Wirral, UK). Pooled proportions for PPTs were calculated using random‐effects models with 95% confidence intervals (CI) as previously published [10]. Heterogeneity between studies was assessed using the Cochran *Q*‐test and the *I* ^2^ statistic, where the *I* ^2^ value indicates the approximate proportion of the total variance between studies that is due to heterogeneity. *I* ^2^ values above 25% indicate moderate heterogeneity, values above 50% indicate large heterogeneity and values above 75% indicate very large heterogeneity. Given the expected high between‐study heterogeneity, pooled proportion analyses were performed using the DerSimonian‐Laird method with random‐effects models [11]. The risk of bias was assessed using the Appraisal tool for cross‐sectional studies (AXIS) tool [12]. To assess the risk of publication bias, Egger's test and funnel plots were conducted. Meta‐analysis was conducted for all possible allergens if the respective allergen was reported in ≥ 2 studies. Subgroup analyses were conducted in patients with AD and according to geographical localisation (Europe, the United States, Asia and the Middle East) for allergens reported in ≥ 2 articles. Sub‐analyses of patients with and without AD were planned but could not be performed due to a limited number of studies. Proportion *Z*‐tests were conducted to compare the pooled proportions of allergens responsible for PPTs in all children to children with AD. Forest plots were constructed to show the direct comparison of data across the studies in the meta‐analysis and the quality of the confidence interval.

## 3 Results

### 3.1 Qualitative Assessment of the Included Studies

#### 3.1.1 Eligible Studies

**FIGURE 1:**
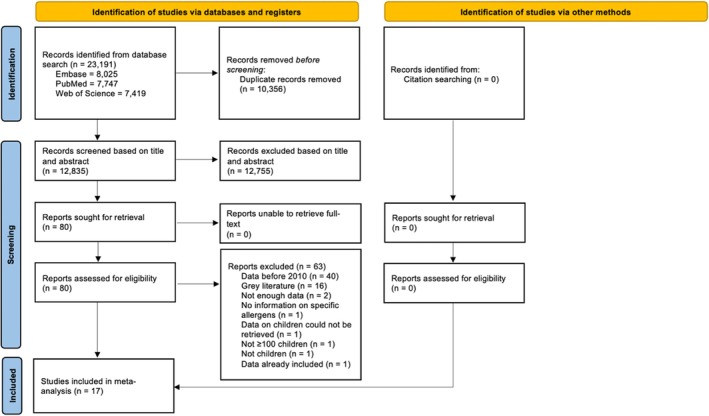
Preferred Items for Systematic Review and Meta‐Analysis flowchart.

In total, 23 191 non‐duplicate articles (PubMed = 7747, Web of Science = 7419, Embase = 8025) were identified through database search. After removing duplicates, 12 835 articles were screened for title and abstract, resulting in a total of 80 articles eligible for full‐text assessment. Of these, 63 articles were excluded for reasons listed in Figure 1. Thus, 17 articles were included for meta‐analysis. No additional articles were included from screening the reference lists of included articles (Figure 1).

#### 3.1.2 Characteristics of the Included Studies

A total of 17 studies published between 2016 and 2024 were included [13, 14, 15, 16, 17, 18, 19, 20, 21, 22, 23, 24, 25, 26, 27, 28, 29]. The studies comprised 11 593 children with 4176 (36%) PPTs. In total, 6705 (57.8%) were girls and the mean age ± standard deviation (SD) was 11.3 ± 2.9 years. Of the included studies, 12 (70.6%) reported the number of patients with AD, yielding a total of 4273 (36.8%) AD patients. In studies on AD reporting the number of PPTs, the total number of PPTs was 1955 (45.7%) (Table 1).

**TABLE 1:** Characteristics of included studies in children.

First author surname (year)	Country	Patients (no.)	Positive patch tests [no. (%)]	Girls [no. (%)]	Age [mean (SD)]	Atopic dermatitis patients [no. (%)]	Positive patch tests in atopic dermatitis patients [no. (%)]
Bonamonte et al. (2022) [29]	Italy	432	185 (42.8)	232 (53.7)	10.4	103 (23.8)	38 (36.9)
Fortina et al. (2016) [13]	Italy	2614	1220 (46.7)	1426 (54.6)	5.3 (2.5)	1283 (49.1)	600 (47.3)
Siemund et al. (2022) [14]	Sweden	196	10 (5.1)	105 (53.6)	9	NA	NA
Lagrelius et al. (2016) [15]	Sweden	2286	349 (15.3)	1199 (52.5)	Median: 16.7 (range: 15.8–18.9)	NA	NA
Machovcová and Machovcova (2012) [16]	Czech Republic	236	106 (44.9)	109 (46.1)	12.6	59 (27.0)	21 (35.6)
Kakşi et al. (2022) [18]	Turkey	191	36 (18.8)	114 (59.7)	12.2 (range: 4–18)	NA	NA
Young et al. (2023) [17]	The United States	612	487 (79.6)	378 (61.7)	11.2 (4.6)	NA	NA
Simonsen et al. (2018) [19]	Denmark	1573	387 (24.5)	1005 (63.9)	12.5 (4.0)	823 (52.3)	188 (22.8)
Johnson et al. (2023) [20]	The United States	912	691 (75.7)	561 (61.5)	11.0 (4.8)	615 (67.4)	483 (78.5)
Boonchai et al. (2021) [21]	Thailand	112	38 (35.5)	75 (67.0)	14.5 (3.2)	26 (23.2)	NA
Handa et al. (2024) [22]	India	136	28 (20.5)	56 (41.1)	5.6 (3.2)	136 (100)	28 (20.5)
Christiansen et al. (2016) [23]	Denmark	249	3 (1.2)^a^	148 (59.4)	14	NA	NA
Jacob et al. (2017) [28]	The United States	552	337 (61.1)	363 (65.8)	10.5 (4.7)	552	337 (61.1)
Andre et al. (2024) [24]	Israel	367	242 (65.9)	234 (63.8)	11.5 (4.14)	116 (31.6)	NA
Slodownik et al. (2023) [25]	Israel	357	153 (42.8)	245 (68.6)	NA	110 (30.8)	40 (37)
Barwari et al. (2023) [26]	The Netherlands	439	334 (76.1)	262 (59.6)	Median: 13 (IQR: 10–16)	271 (61.7)	212 (78.2)
Noë et al. (2022) [27]	Belgium	329	119 (36)	193 (59)	10.9	179 (54.4)	78 (43.5)

Abbreviations: IQR, interquartile range; NA, Not Applicable.

^a^ Only investigated nickel sulphate 200 μg/cm^2^.

Three studies were conducted in the United States [17, 20, 28], two each in Italy [13, 29], Sweden [14, 15], Israel [24, 25] and Denmark [19, 23], and one each in Czech Republic [16], Turkey [18], Thailand [21], India [22], The Netherlands [26] and Belgium [27]. The risk of bias was generally low/acceptable according to the AXIS tool (Table S1).

### 3.2 Quantitative Assessment of the Included Studies

#### 3.2.1 Allergens in All Children

**FIGURE 2:**
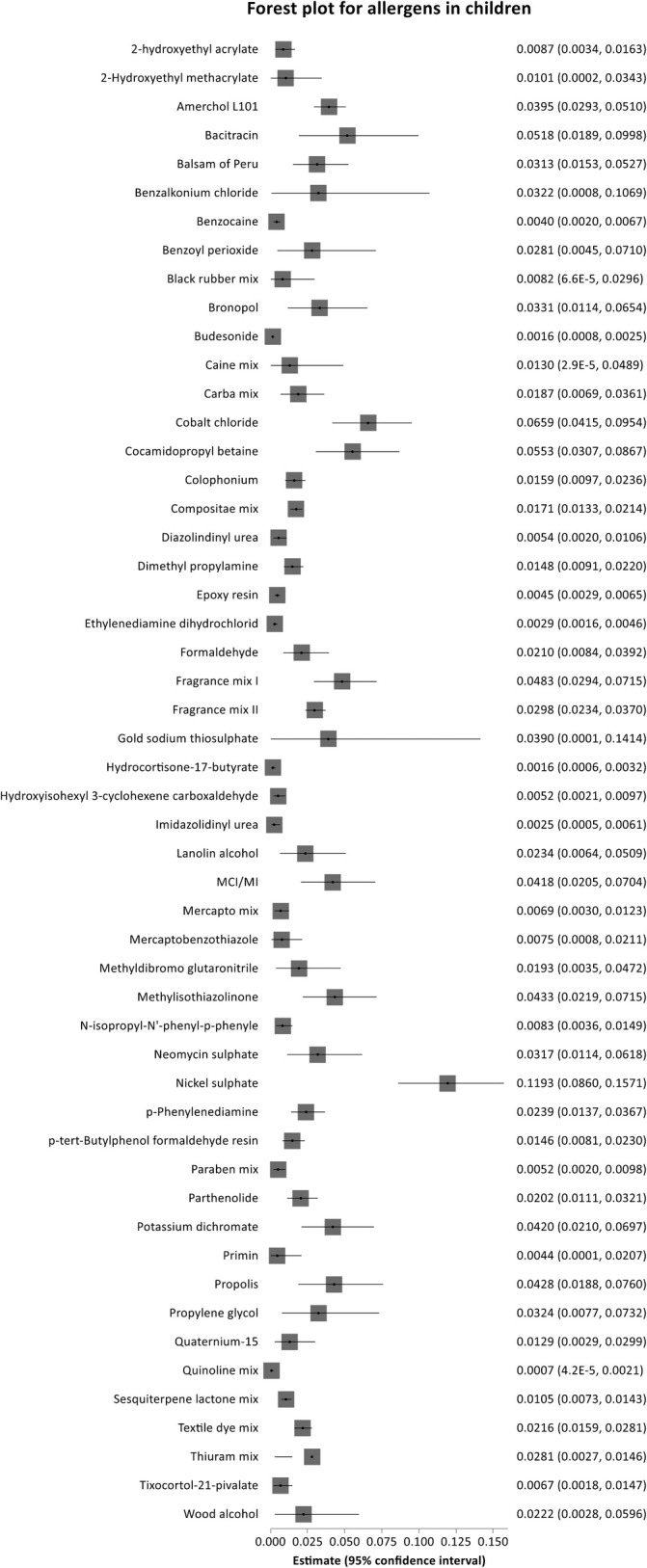
Forest plot for allergens and corresponding proportions in all children.

Among the 17 included articles, a total of 52 allergens were reported in ≥ 2 articles, thus allowing for meta‐analysis. Of these, 8 (15.4%) allergens were tested in at least 10 studies each. There were 35 (67.3%) allergens with a pooled prevalence of PPTs above 1% (Table 2). The proportion of PPTs of allergens reported in at least two studies is shown in Table 2. Among the 52 allergens, 17 (32.7%) had a proportion below 1% (Table 2 and Figure 2). Generally, the between‐study heterogeneity of the included studies was high based on *Q*‐test and *I* ^2^ (Table 2).

**TABLE 2:** Allergens responsible for positive patch tests in all children.

Allergen	Studies [*n*]	Proportion of positive reactions [%]	Lower 95% CI	Upper 95% CI	*p* for *Q*‐test	*I* ^2^ (95% CI) [%]
Nickel sulphate	16	11.9	8.6	15.7	< 0.0001	96.9 (96.2–97.4)
Cobalt chloride	14	6.6	4.2	9.5	< 0.0001	96.7 (95.9–97.2)
Cocamidopropyl betaine	3	5.5	3.1	8.7	< 0.0001	91.9 (76.8–95.7)
Bacitracin	2	5.2	1.2	10.0	0.0005	91.7 (NA)
Fragrance mix I	13	4.8	2.9	7.1	< 0.0001	94.5 (92.7–95.7)
MI	8	4.3	2.2	7.2	< 0.0001	93.6 (90.2–95.4)
Propolis	3	4.3	1.9	7.6	0.0001	88.8 (58.7–94.5)
Potassium dichromate	13	4.2	2.1	7.0	< 0.0001	97 (96.4–97.5)
MCI/MI	10	4.2	2.0	7.0	< 0.0001	96.2 (95–97)
Amerchol L101	2	3.9	2.9	5.1	< 0.0001	94.1 (NA)
Gold sodium thiosulphate	2	3.9	0.01	14.1	< 0.0001	96.5 (NA)
Bronopol	4	3.3	1.1	6.5	< 0.0001	93.5 (86.9–96)
Propylene glycol	5	3.2	0.8	7.3	< 0.0001	96.8 (95.2–97.6)
Benzalkonium chloride	2	3.2	0.08	10.7	< 0.0001	93.4 (NA)
Neomycin sulphate	9	3.2	1.1	6.2	< 0.0001	97.5 (96.9–98)
Balsam of Peru	11	3.1	1.5	5.3	< 0.0001	96.1 (94.9–96.9)
Fragrance mix II	8	3.0	2.3	3.7	0.1	40.1 (0–72.1)
Benzoyl peroxide	3	2.8	0.4	7.1	0.0001	89 (59.9–94.6)
Thiuram mix	7	2.8	0.3	1.4	< 0.0001	82 (59.1–89.6)
p‐Phenylenediamine	7	2.4	1.4	3.7	< 0.0001	82.2 (59–9‐89.7)
Lanolin alcohol	7	2.3	0.6	5.0	< 0.0001	96.1 (94.5–97.1)
p‐tert‐Butylphenol formaldehyde resin	8	1.5	0.8	2.3	< 0.0001	80.3 (57–88.4)
Wood alcohol	3	2.2	0.3	6.0	0.0002	88.3 (54.7–94.3)
Textile dye mix	6	2.2	1.6	2.8	0.4	0.8 (0–61.3)
Formaldehyde	12	2.1	0.8	3.9	< 0.0001	95.3 (93.8–96.3)
Parthenolide	2	2.0	1.1	3.2	0.8	0 (NA)
Methyldibromo glutaronitrile	3	1.9	0.4	4.7	0.0005	86.8 (42.2–93.8)
Carba mix	5	1.9	0.7	3.6	< 0.0001	93.1 (97.3–95.5)
Compositae mix	5	1.7	1.3	2.1	0.4	4.8 (0–65.8)
Colophonium	12	1.6	1.0	2.4	< 0.0001	83.8 (72.5–89.2)
Dimethyl propylamine	2	1.5	0.9	2.2	1.0	0 (NA)
Caine mix	3	1.3	0.003	4.9	< 0.0001	95 (89.1–97)
Quaternium‐15	7	1.3	0.3	3.0	< 0.0001	94.9 (92.3–96.3)
Sesquiterpene lactone mix	5	1.0	0.7	1.4	0.4	0 (0–64.1)
2‐Hydroxyethyl methacrylate	2	1.0	0.02	3.4	0.01	83.2 (NA)
2‐Hydroxyethyl acrylate	2	0.9	0.3	1.6	0.8	0 (NA)
N‐isopropyl‐N′‐phenyl‐p‐phenylenediamine	6	0.8	0.4	1.5	0.006	69.4 (0–85)
Black rubber mix	3	0.8	0.007	3.0	< 0.0001	89.2 (61.4–94.7)
Mercaptobenzothiazole	6	0.7	0.08	2.1	< 0.0001	95.3 (92.8–96.6)
Mercapto mix	6	0.7	0.3	1.2	0.03	58.9 (0–81.3)
Tixocortol‐21‐pivalate	6	0.7	0.2	1.5	< 0.0001	86.7 (71.3–92.2)
Diazolindinyl urea	5	0.5	0.2	1.0	0.01	68.9 (0–85.8)
Paraben mix	7	0.5	0.2	1.0	0.0001	77.9 (44.7–87.8)
Hydroxyisohexyl 3‐cyclohexene carboxaldehyde (Lyral)	3	0.5	0.2	1.0	0.3	12.8 (0–76.2)
Epoxy resin	6	0.5	0.3	0.7	0.3	16.7 (0–67.2)
Primin	2	0.4	0.01	2.0	0.04	77 (NA)
Benzocaine	4	0.4	0.2	0.7	0.3	18.1 (0–73.3)
Ethylenediamine dihydrochloride	2	0.3	0.2	0.6	0.6	0 (NA)
Imidazolidinyl urea	4	0.3	0.05	0.6	0.04	64 (0–85.7)
Hydrocortisone‐17‐butyrate	4	0.2	0.06	0.3	0.5	0 (0–67.9)
Budesonide	6	0.2	0.08	0.3	0.4	0 (0–61)
Quinoline mix	2	0.07	0.004	0.2	0.8	0 (NA)

Abbreviations: CI, confidence interval; MCI/MI, Methylchloroisothiazolinone/methylisothiazolinone; MI, methylisothiazolinone; n, number; NA, not available.

##### 3.2.1.1 PPTs to Metals in All Children

According to our findings, the allergen with the highest proportion of PPTs was nickel sulphate (nickel), yielding 11.9% (95% CI, 8.6%–15.7%) based on 16 studies [13, 15, 16, 17, 18, 19, 20, 21, 22, 23, 24, 25, 26, 27, 28, 29] with high between‐study heterogeneity (*I* ^2^ = 96.9%) (Table 2 and Figure S1). Cobalt chloride (cobalt) showed a pooled proportion of 6.6% (95% CI, 4.2%–9.5%) based on 14 studies [13, 15, 16, 17, 18, 19, 20, 21, 24, 25, 26, 27, 28, 29]. Among all children, the pooled proportion of PPTs to potassium dichromate (chromium) was 4.2% (95% CI, 2.1%–7.0%) based on 13 studies [13, 15, 16, 17, 19, 21, 22, 24, 25, 26, 27, 28, 29]. Regarding gold sodium thiosulphate (Gold), the pooled prevalence of PPTs was 3.9% (95% CI, 0.01%–14.1%) based on two studies [17, 27] (Table 2).

##### 3.2.1.2 PPTs to Perfumes in All Children

Meta‐analyses of the following perfume allergens were conducted: fragrance mix I (FM I), fragrance mix II (FM II), hydroxyisohexyl 3‐cyclohexene carboxaldehyde (HICC) and Balsam of Peru.

FM I was reported in 13 studies [15, 17, 18, 19, 20, 21, 22, 24, 25, 26, 27, 28, 29] exhibiting a proportion of PPTs of 4.8% (95% CI, 2.9%–7.1%) with substantial between‐study heterogeneity (*I* ^2^ = 94.5%). FM II revealed a lower rate of PPTs of 3.0% (95% CI, 2.3%–3.7%) based on eight studies [19, 20, 21, 24, 26, 27, 28, 29] with *I* ^2^ = 40% suggesting more consistent findings between studies. The proportion of PPTs to HICC was 0.5% (95% CI, 0.2%–1.0%) based on three studies [19, 21, 26] with low between‐study heterogeneity (*I* ^2^ = 12.8%). For Balsam of Peru, the pooled proportion of PPTs was 3.1% (95% CI, 1.5%–5.3%) based on 11 studies [13, 15, 17, 18, 19, 20, 21, 22, 26, 28, 29] with high between‐study heterogeneity (*I* ^2^ = 96.1%) (Table 2).

##### 3.2.1.3 PPTs to Preservatives in All Children

The following preservatives allowed for pooled analyses: Methylchloroisothiazolinone/Methylisothiazolinone (MCI/MI), MI as a stand‐alone preservative, paraben mix, formaldehyde and the two formaldehyde releasers bronopol and diazolindinyl urea.

MI revealed a rate of PPTs of 4.3% (95% CI, 2.2%–7.2%) based on eight studies [19, 20, 21, 25, 26, 27, 28, 29] with substantial between‐study heterogeneity (*I* ^2^ = 93.6%). Similarly, the proportion of PPTs for MCI/MI was 4.2% (95% CI: 2.0%–7.0%) based on 10 studies [15, 16, 17, 19, 20, 21, 24, 25, 26, 28]. For bronopol, the rate of PPTs was 3.3% (95% CI, 1.1%–6.5%) based on four studies [17, 19, 20, 28] with high between‐study heterogeneity (*I* ^2^ = 93.5%). PPTs to formaldehyde were reported in 12 studies [15, 16, 17, 18, 19, 20, 21, 24, 26, 27, 28, 29], yielding a pooled proportion of 2.1% (95% CI, 0.8%–3.9%). Diazolindinyl urea and paraben mix both revealed low rates of PPTs of 0.5% (95% CI, 0.2%–1.0%) and 0.5% (95% CI, 0.2%–1.0%), respectively. This was based on five studies [15, 19, 20, 21, 25] for diazolindinyl urea and seven studies [13, 15, 19, 20, 21, 22, 29] for paraben mix (Table 2).

##### 3.2.1.4 Pooled Prevalences According to Geographical Regions in All Children

Among 52 allergens reported in ≥ 2 articles, only 4 (7.7%) were reported in all four geographical regions of interest (Europe, the United States, Asia and the Middle East), and pooled prevalence of 41 allergens (78.8%) could be calculated according to at least one geographical region (Table 3).

**TABLE 3:** Comparative pooled prevalences according to geographical localisation in all children.

	Europe		United States		Asia		Middle East	
Allergen	Studies [*n*]	Proportion of positive reactions (95% CI) [%]	Studies [*n*]	Proportion of positive reactions (95% CI) [%]	Studies [*n*]	Proportion of positive reactions (95% CI) [%]	Studies [*n*]	Proportion of positive reactions (95% CI) [%]
Nickel sulphate	8	**10.2 (5.4–16.2)**	3	**18.0 (14.6–21.9)**	2	7.6 (1.5–17.9)	3	14.1 (10.5–18.0)
Cobalt chloride	8	5.4 (4.9–6.0)	2	9.4 (8.0–10.9)	NA	NA	2	4.5 (2.9–6.3)
Cocamidopropyl betaine	NA	NA	2	7.0 (5.7–8.3)	NA	NA	NA	NA
Bacitracin	NA	NA	2	5.2 (1.9–10.0)	NA	NA	NA	NA
Fragrance mix I	5	3.7 (1.9–6.0)	3	10.9 (9.6–12.3)	2	48.9 (2.5–7.9)	3	3.0 (1.9–4.4)
MI	4	2.9 (2.2–3.5)	2	6.7 (5.4–8.0)	NA	NA	NA	NA
Propolis	NA	NA	2	5.9 (4.8–7.2)	NA	NA	NA	NA
Potassium dichromate	7	3.7 (3.3–4.2)	2	4.1 (3.0–5.3)	2	5.7 (3.1–9.0)	2	3.3 (2.1–4.8)
MCI/MI	4	1.4 (1.1–1.8)	3	6.9 (5.9–8.0)	NA	NA	2	4.7 (3.3–6.3)
Gold sodium thiosulphate	NA	NA	NA	NA	NA	NA	NA	NA
Bronopol	NA	NA	3	4.8 (3.9–5.8)	NA	NA	NA	NA
Propylene glycol	2	0.5 (0.08–1.4)	3	5.9 (4.9–7.0)	NA	NA	NA	NA
Benzalkonium chloride	NA	NA	NA	NA	NA	NA	NA	NA
Neomycin sulphate	5	1.7 (1.4–2.0)	NA	NA	NA	NA	NA	NA
Balsam of Peru	5	1.5 (1.2–1.8)	3	8.3 (7.2–9.6)	2	2.0 (0.6–4.1)	NA	NA
Fragrance mix II	4	2.6 (1.7–3.6)	2	3.4 (2.5–4.4)	NA	NA	NA	NA
Benzoyl peroxide	NA	NA	NA	NA	NA	NA	NA	NA
Thiuram mix	4	0.7 (0.3–1.4)	NA	NA	NA	NA	NA	NA
p‐Phenylenediamine	4	1.7 (1.0–2.6)	NA	NA	NA	NA	2	4.5 (1.8–8.3)
Lanolin alcohol	4	1.9 (0.4–4.6)	NA	NA	NA	NA	NA	NA
p‐tert‐Butylphenol formaldehyde resin	3	0.7 (4.1–1.1)	NA	NA	NA	NA	NA	NA
Wood alcohol	NA	NA	NA	NA	NA	NA	NA	NA
Textile dye mix	4	2.1 (1.4–2.8)	NA	NA	NA	NA	2	2.2 (0.8–4.4)
Formaldehyde	6	0.7 (0.4–1.2)	3	7.0 (6.0–8.0)	NA	NA	2	1.7 (0.3–4.5)
Parthenolide	NA	NA	NA	NA	NA	NA	NA	NA
Methyldibromo glutaronitrile	2	2.0 (0.1–6.1)	NA	NA	NA	NA	NA	NA
Carba mix	3	1.0 (0.2–2.3)	NA	NA	NA	NA	NA	NA
Compositae mix	2	1.5 (1.1–2.0)	2	2.2 (1.5–3.0)	NA	NA	NA	NA
Colophonium	6	1.5 (0.7–2.7)	2	1.9 (0.4–4.5)	2	1.9 (0.4–4.5)	2	1.4 (0.5–2.6)
Dimethyl propylamine	NA	NA	NA	NA	NA	NA	NA	NA
Caine mix	3	1.3 (0.003–5.0)	NA	NA	NA	NA	NA	NA
Quaternium‐15	4	0.52 (0.046–1.5)	2	3.5 (1.4–6.5)	NA	NA	NA	NA
Sesquiterpene lactone mix	3	4.8 (3.9–5.8)	NA	NA	NA	NA	NA	NA
2‐Hydroxyethyl methacrylate	NA	NA	NA	NA	NA	NA	NA	NA
2‐Hydroxyethyl acrylate	NA	NA	NA	NA	NA	NA	NA	NA
N‐isopropyl‐N′‐phenyl‐p‐phenylenediamine	4	0.7 (0.3–1.4)	NA	NA	NA	NA	NA	NA
Black rubber mix	2	0.5 (0.06–3.0)	NA	NA	NA	NA	NA	NA
Mercaptobenzothiazole	5	0.8 (0.08–2.4)	NA	NA	NA	NA	NA	NA
Mercapto mix	3	0.6 (0.2–1.3)	NA	NA	2	1.3 (0.2–3.1)	NA	NA
Tixocortol‐21‐pivalate	3	0.2 (0.1–0.4)	2	1.9 (0.4–4.5)	NA	NA	NA	NA
Diazolindinyl urea	2	0.3 (0.1–0.5)	NA	NA	NA	NA	NA	NA
Paraben mix	4	0.3 (0.1–0.5)	NA	NA	2	1.2 (0.2–2.9)	NA	NA
Hydroxyisohexyl 3‐cyclohexene carboxaldehyde (Lyral)	2	0.6 (0.1–1.3)	NA	NA	NA	NA	NA	NA
Epoxy resin	5	0.5 (0.3–0.7)	NA	NA	NA	NA	NA	NA
Primin	NA	NA	NA	NA	NA	NA	NA	NA
Benzocaine	3	0.4 (0.2–0.6)	NA	NA	NA	NA	NA	NA
Ethylenediamine dihydrochloride	NA	NA	NA	NA	NA	NA	NA	NA
Imidazolidinyl urea	2	0.1 (0.02–0.3)	NA	NA	NA	NA	NA	NA
Hydrocortisone‐17‐butyrate	2	0.1 (0.2–0.3)	NA	NA	NA	NA	NA	NA
Budesonide	4	0.1 (0.06–0.2)	NA	NA	NA	NA	NA	NA
Quinoline mix	2	0.07 (0.004–0.2)	NA	NA	NA	NA	NA	NA

*Note:* Bold text marks the importance of nickel regulation.

Abbreviations: CI, confidence interval; MCI/MI, Methylchloroisothiazolinone/methylisothiazolinone; MI, methylisothiazolinone; n, number; NA, not available.

For nickel, the pooled prevalence could be calculated for all regions, yielding proportions of 10.2% (95% CI, 5.4%–16.2%) in Europe, 18.0% (95% CI, 14.6%–21.9%) in the United States, 7.6% (95% CI, 1.5%–17.9%) in Asia and 14.1% (95% CI, 10.5%–18.0%) in the Middle East, respectively. For cobalt, the pooled proportion of PPTs was 5.4% (95% CI, 4.9%–6.0%) in Europe, 9.4% (95% CI, 8.0%–10.9%) in the United States and 4.5% (95% CI, 2.9%–6.3%) in the Middle East (Table 3).

Interestingly, the pooled prevalence of FM I was 3.7% (95% CI, 1.9%–6.0%) in Europe based on five studies [15, 19, 26, 27, 29], 10.9% (95% CI, 9.6%–12.3%) in the US based on three studies [17, 20, 28] and 3.0% (95% CI, 1.9%–4.4%) in the Middle East based on three studies [18, 24, 25]. In Asia, the pooled prevalence was 4.9% (95% CI, 2.5%–7.9%) based on two studies [21, 22] (Table 3).

For MI, only pooled prevalences could be conducted for Europe and the United States. For Europe, the pooled proportion of PPTs was 2.9% (95% CI, 2.2%–3.5%) based on four studies [19, 25, 26, 27] and 6.7% (95% CI, 5.4%–8.0%) for the US based on two studies [20, 28]. Pooled prevalences of PPTs to MCI/MI could be calculated in Europe, the United States and the Middle East, yielding estimates of 1.4% (95% CI, 1.1%–1.8%), 6.9% (95% CI, 5.9%–8.0%) and 4.7% (95% CI, 3.3%–6.3%), respectively. These findings were based on four studies in Europe [15, 16, 19, 26], three studies in the United States [17, 20, 28] and two studies in the Middle East [24, 25]. For propylene glycol, the pooled proportion of PPTs in Europe was 0.5% (95% CI, 0.08%–1.4%) based on two studies [13, 27] and 5.9% (95% CI, 4.9%–7.0%) in the US based on three studies [17, 20, 28] (Table 3).

#### 3.2.2 Allergens in Children With Atopic Dermatitis

Of the 12 studies [13, 16, 19, 20, 21, 22, 24, 25, 26, 27, 28, 29] reporting data on children with AD, only 10 [13, 16, 19, 20, 22, 25, 26, 27, 28, 29] reported the number of PPTs of specific allergens, thus being eligible for meta‐analysis. A total of 70 allergens were reported in the eight studies, of which 43 (61.4%) allergens were reported in ≥ 2 studies and thus included in the meta‐analysis. Of these, only 2 (4.6%) allergens [nickel and cobalt] were reported in all eight studies. Furthermore, 19 allergens (44.2%) were reported in only two studies each (Table 4).

**TABLE 4:** Allergens responsible for positive patch tests in children with atopic dermatitis.

Allergen	Studies [*n*]	Proportion of positive reactions [%]	Lower 95% CI	Upper 95% CI	*p* for *Q*‐test	*I* ^2^ (95% CI) [%]
Nickel sulphate	8	12.6	7.8	18.4	< 0.0001	93.5 (90–1.95.4)
Cocamidopropyl betaine	2	9.0	7.3	10.8	0.6	0 (NA)
Cobalt chloride	6	8.2	4.8	12.5	< 0.0001	90.8 (82.9–94.1)
Fragrance mix I	7	6.6	3.5	10.7	< 0.0001	91.9 (86.4–94.6)
Propylene glycol	2	6.6	4.6	8.8	0.2	48.8 (NA)
Bacitracin	2	5.9	3.0009	9.6	0.02	83.1 (NA)
MCI/MI	5	6.0	2.6	8.1	< 0.0001	88.9 (75.2–93.5)
Potassium dichromate	6	4.7	2.2	8.05	< 0.0001	87.5 (73.6–92.5)
MI	6	4.7	1.8	8.9	< 0.0001	93.3 (88.6–95.5)
Lanolin alcohol	2	4.2	0.2	13.08	< 0.0001	95.6 (NA)
Neomycin sulphate	6	4.2	1.6	7.9	< 0.0001	92.3 (86.4–94.9)
Balsam of Peru	6	3.7	1.4	7.0	< 0.0001	92.2 (86.2–94.9)
Fragrance mix II	5	3.6	2.3	5.2	0.02	66.9 (0–85.2)
Carba mix	2	3.5	1.7	5.9	0.1	58.9 (NA)
Wood alcohol	2	3.02	0.2	9.0	0.006	86.9 (NA)
Bronopol	3	3.0	1.1	5.8	0.001	85.5 (30–93.4)
Formaldehyde	5	2.6	0.5	6.3	< 0.0001	94.4 (90.5–96.3)
Propolis	2	2.6	0.1	8.08	0.0004	92 (NA)
Compositae mix	2	2.6	1.7	3.6	0.3	0 (NA)
Parthenolide	2	2.06	1.1	3.3	0.8	0 (NA)
Quaternium‐15	4	2.05	0.5	4.6	< 0.0001	91.4 (80.3–95)
Dimethyl propylamine	2	1.8	1.0	2.9	0.7	0 (NA)
Methyldibromo glutaronitrile	2	1.7	0.2	4.6	0.02	83 (NA)
Caine mix	2	1.7	0.08	8.06	0.003	88.3 (NA)
Colophonium	7	1.5	0.9	2.3	0.09	45.6 (0–75.4)
Textile dye mix	3	1.4	0.6	2.4	0.8	0 (0–72.9)
p‐Phenylenediamine	4	1.2	0.5	2.2	0.3	25.3 (0–75.3)
Sesquiterpene lactone mix	3	1.1	0.7	1.7	0.4	0 (0–72.9)
Tixocortol‐21‐pivalate	4	1.04	0.2	2.7	< 0.0001	88.1 (67–93.6)
Mercapto mix	2	1.02	0.1	2.8	0.02	76 (0–90.7)
Black rubber mix	2	1.0	0.2	2.3	0.3	7.6 (NA)
Paraben mix	4	1.0	0.3	2.08	0.04	64.5 (0–85.8)
N‐isopropyl‐N′‐phenyl‐p‐phenylenediamine	3	0.9	0.05	2.9	0.01	76.3 (0–90.7)
Thiuram mix	4	0.8	0.2	1.7	0.04	64.1 (0–85.7)
p‐tert‐Butylphenol formaldehyde	4	0.7	0.4	1.2	0.3	13.1 (0–71.9)
Diazolindinyl urea	2	0.7	0.2	1.5	0.1	53.1 (NA)
Hydroxyisohexyl 3‐cyclohexene carboxaldehyde	2	0.6	0.06	1.8	0.1	62.8 (NA)
Benzocaine	3	0.5	0.1	1.09	0.7	0 (0–72.9)
Imidazolidinyl urea	2	0.5	0.2	0.9	0.6	0 (NA)
2‐Hydroxyethyl methacrylate	2	0.4	0.03	1.4	0.7	0 (NA)
Epoxy resin	3	0.4	0.05	1.2	0.2	36.8 (0–81.7)
Mercaptobenzothiazole	2	0.4	0.1	0.9	0.7	0 (NA)
Budesonide	3	0.2	0.02	0.4	0.4	0 (0–72.9)

Abbreviations: CI, confidence interval; MCI/MI, Methylchloroisothiazolinone/methylisothiazolinone; MI, methylisothiazolinone; n, number; NA, not available.

**FIGURE 3:**
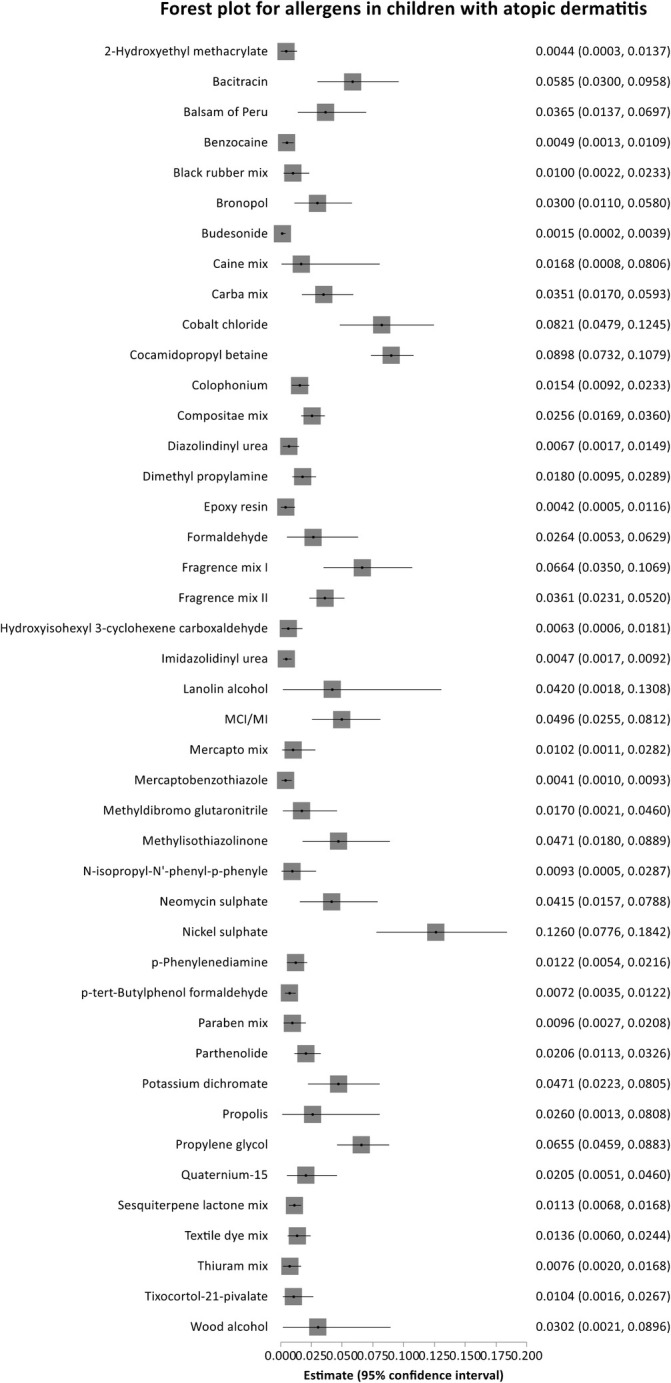
Forest plot for allergens and corresponding proportions in children with atopic dermatitis.

Like the main analysis, nickel showed the highest proportion of PPTs in children with AD, yielding a pooled prevalence of 12.6% (95% CI, 7.8%–18.4%) based on eight studies [16, 19, 20, 22, 25, 26, 28, 29] (Table 3 and Figure S2). Other than nickel, the allergens with the highest pooled proportions of PPTs were cocamidopropyl betaine, cobalt and FM I (Figure 3). Cocamidopropyl betaine was reported in two studies [20, 28] with a proportion of 9.0% (95% CI, 7.3%–10.8%). Cobalt was reported in six studies [19, 20, 25, 26, 28, 29], yielding a proportion of PPTs of 8.2% (95% CI, 4.8%–12.5%), whereas FM I was reported in seven studies [19, 20, 22, 25, 26, 28, 29] with a pooled proportion of PPTs of 6.6% (95% CI, 4.8%–12.5%). The between‐study heterogeneity of the included studies on children with AD was high for most allergens (Table 4).

##### 3.2.2.1 Allergens in All Children Versus Children With Atopic Dermatitis

Based on the findings above, a *Z*‐test was conducted to reveal differences in the proportion of PPTs in all children compared to children with AD. A total of 43 allergens were included in the analysis, of which 6 (13.9%) were found to be significantly different. The results are shown in Table 5.

**TABLE 5:** Comparison analysis of all children versus children with atopic dermatitis.

Allergen	Proportion of positive reactions, all children [%]	Proportion of positive reactions, AD children [%]	*p*
Cocamidopropyl betaine	5.5	9.0	**0.0030**
Neomycin sulphate	3.2	4.2	0.2441
Propylene glycol	3.2	6.6	**0.0006**
Textile dye mix	2.2	1.4	0.1783
MI	4.3	4.7	0.6829
Hydroxyisohexyl 3‐cyclohexene carboxaldehyde	0.5	0.6	0.7491
Tixocortol‐21‐pivalate	0.7	1.0	0.3690
Mercaptobenzothiazole	0.8	0.4	0.3144
N‐isopropyl‐N′‐phenyl‐p‐phenylenediamine	0.8	0.9	0.8082
Colophonium	1.6	1.5	0.9381
Caine mix	1.3	1.7	0.4741
Wood alcohol	2.2	3.0	0.2655
Nickel sulphate	11.9	12.6	0.6443
Thiuram mix	2.8	0.8	**0.0005**
Dimethyl propylamine	1.5	1.8	0.5847
Bronopol	3.3	3.0	0.6948
Budesonide	0.2	0.2	0.9698
p‐tert‐Butylphenol formaldehyde resin	1.5	0.7	0.91
Quaternium‐15	1.3	2.1	0.1816
Lanolin alcohol	2.3	4.2	**0.0198**
Formaldehyde	2.1	2.6	0.4332
Potassium dichromate	4.2	4.7	0.5783
Methyldibromo glutaronitrile	1.9	1.7	0.7088
Bacitracin	5.2	5.9	0.5114
Propolis	4.3	2.6	**0.0395**
MCI/MI	4.2	5.0	0.4049
Diazolindinyl urea	0.5	0.7	0.7133
Mercapto mix	0.7	1.0	0.4185
Benzocaine	0.4	0.5	0.7691
Balsam of Peru	3.1	3.7	0.5196
Black rubber mix	0.8	1.0	0.6745
Imidazolidinyl urea	0.3	0.5	0.4289
Paraben mix	0.5	1.0	0.2524
Parthenolide	2.0	2.1	0.9555
Carba mix	1.9	3.5	**0.0235**
p‐Phenylenediamine	2.4	1.2	0.0495
Sesquiterpene lactone mix	1.1	1.1	0.8730
Epoxy resin	0.5	0.4	0.9183
2‐Hydroxyethyl methacrylate	1.0	0.4	0.1326
Cobalt chloride	6.6	8.2	0.1656
Compositae mix	1.7	2.6	0.1897
Fragrance mix I	4.8	6.6	49.18
Fragrance mix II	3.0	3.6	40.60

*Note: p* < 0.05 are marked in bold.

Abbreviations: AD, atopic dermatitis; MI, methylisothiazolinone.

According to our findings, children with AD had a significantly higher proportion of PPTs for cocamidopropyl betaine (9.0% vs. 5.5%, *p* = 0.0030), propylene glycol (6.6% vs. 3.2%, *p* = 0.0006), lanolin alcohol (4.2% vs. 2.3%, *p* = 0.019) and carba mix (3.5% vs. 1.9%, *p* = 0.023) compared to all children. All children had a higher proportion of PPTs for thiuram mix (2.8% vs. 0.8%, *p* = 0.0005) and propolis (4.3% vs. 2.6%, *p* = 0.039) compared to children with AD. For nickel, the allergen with the highest proportion in both all children and children with AD, no significant difference was found (Table 5).

## 4 Discussion

In this systematic review and meta‐analysis, we found the most prevalent allergen to be nickel in both ‘all children’ and ‘children with AD’, followed by cobalt. Furthermore, significant differences in the proportion of PPTs were seen between the two populations for cocamidopropyl betaine, propylene glycol, thiuram mix, lanolin alcohol, propolis and carba mix.

### 4.1 Metals

Nickel has been regulated in Denmark since 1990 [30], and in 1991, the content of nickel in ear‐piercing posts and earrings in Sweden was regulated [31]. With the introduction of the European Union (EU) Nickel Directive in 1994 with a full implementation in 2001 [6], the prevalence of CA due to nickel has decreased [7, 32, 33]. In our study, we found a slightly lower prevalence of nickel allergy in children compared to Bonitsis et al. in 2011 (11.9% vs. 14.5%) [5]. In a Danish study from 2018, Simonsen et al. reported a significant decrease in CA to nickel during the periods 2003–2011 and 2012–2016, with rates declining from 9.7% to 7.0% in 2587 and 1540 patch‐tested children, respectively [19]. The prominence of nickel as the leading allergen with the highest proportion of PPTs aligns with existing literature [18, 29], highlighting its ubiquitous presence in various consumer products including jewellery, clothing fasteners and household items [34] and the need to revisit the effectiveness of the current EU regulation, as recently suggested [35]. In the current study, major geographical differences were observed, highlighting the need for regulations in the United States like those in Europe.

Cobalt revealed the second‐highest pooled proportion of PPTs at 6.6%. However, prevalence rates of CA to cobalt in children have been reported with considerable variability. Our results note a slight increase from the study by Bonitsis et al. with a pooled prevalence of 5.9% [5]. On the other hand, a recent study of children patch tested with cobalt by the North American Contact Dermatitis Group between 2001 and 2018 revealed that the prevalence of PPTs to cobalt was 11.9% [36]. Conversely, other studies have found a lower prevalence in paediatric populations. These differences may partly be explained by geographical differences and variations in sample sizes and diagnostic criteria for exposure and sensitisation to cobalt. The findings in the current study underline the need for regulatory limits for cobalt.

In May 2015, an EU regulation was implemented restricting the content of hexavalent chromium to no more than 3 ppm in leather products encountering the skin [37]. In our study, we noted a slight increase in the prevalence of CA to chromium as compared to the previous study by Bonitsis et al. [5] (4.2% vs. 2.9%), including an estimate of 3.7% in Europe. This might partly be explained by the inclusion of study populations from before 2015, differing chromium exposure patterns in children and/or inadequacy of current regulatory actions.

We found a pooled prevalence of PPTs to gold of 3.9% based on two studies. Patch tests have frequently yielded elevated positivity rates for gold, thus rendering the evaluation of clinical reliability a significant challenge. A recent systemic review and meta‐analysis found pooled prevalences of PPTs to gold of 14.1% based on 14 887 dermatitis patients [38]. Despite the high number of PPTs, it has been indicated that PPTs to gold are not a reliable indicator of clinically relevant allergic reactions to daily gold exposure due to the high number of PPTs in routine patch testing [39, 40]. Moreover, our results only rely on two studies and should therefore be interpreted with caution. With this in mind, we do not consider gold to be a relevant allergen to include in routine patch testing of children.

### 4.2 Fragrances

We found the pooled prevalence of PPTs to FM I of 4.8% and 3% for FM II in all children. This is a slight increase compared to the pooled proportion of PPTs of 4% for FM I presented by Bonitsis et al. [5] The current study highlights that CA to FM I and FM II remains high with higher proportions of PPTs to FM I than FM II, in accordance with previous studies [41, 42, 43]. Moreover, large geographical differences of PPTs to FM I were found in Europe (3.7% [95% CI, 1.9%–6.0%]) and the United States (10.9% [95% CI, 9.6%–12.3%]). Several factors may influence these estimates. Importantly, the estimates were based on only five and three studies, respectively, thus challenging extrapolation of the results. Secondly, heterogeneous regulations on fragrance allergens between the two regions may explain these differences. Recently, EU regulations have sought to reduce exposure to prevalent allergens [44], whereas similar regulations have not been considered in the United States.

### 4.3 Preservatives

Preservatives are necessary to prevent deterioration and spoilage caused by microbial growth in water‐based products like cosmetic products, but their use poses a risk to the consumer regardless of age. The cosmetic products that children are in skin contact with are presumably soaps, shampoo, balsams, lotions, creams, deodorants, perfumes and wet wipes.

We observed a notable geographical difference in the pooled prevalence of MI and MCI/MI between Europe and the United States, with prevalences more than double for the United States compared to Europe (MI: 6.7% vs. 2.9%; MCI/MI: 6.9% vs. 1.4%). This aligns with a recently published study by Schwensen et al., which investigated the prevalence of CA to MI in Europe and reported a prevalence of 2.9% [45], highlighting a decrease in the prevalence of CA to MI compared to its two predecessors [46, 47]. However, our findings mark an increase compared to the pooled prevalence of MCI/MI of 1.2% in the study by Bonitsis et al. [5]. A recent study suggested a decrease in CA to MI in Europe and an increase in North America [48]. These geographical differences may primarily be driven by the lack of formal regulation of MCI/MI and MI in the United States compared to stringent regulations in Europe.

Another explanation could be that most of the period covered by the data in the study by Bonitsis et al. [5] predates the use of MI in cosmetic products in 2004 and onwards in the EU [49, 50]. The use of MI in cosmetic products including wet wipes may be the main source of exposure to MI and MCI/MI in children as for adults [47]. The early signs of an epidemic of MI CA were not recognised until 2010, following the increased use of MI in cosmetic products due to the patent loss of Kathon (MCI/MI) around the millennium and the approval of MI as a stand‐alone preservative in cosmetic products in the EU [46, 51] in, respectively, March 2003 [49] and April 2004 [50]. These observations align with a well‐known phenomenon: it takes years from the legal approval of a substance for the industry to begin using it, for consumer exposure patterns to shift and for scientific and regulatory authorities to recognise and understand the subsequent increase in reports of CA cases. Since the height of the MI CA epidemic from the early 2010s to mid‐2010s, MI has been banned for use in leave‐on cosmetics, and its use in rinse‐off cosmetics products has been restricted [52, 53], a significant decrease in the prevalence of MI CA has started to show across the EU [45, 46, 47]. However, this also means that the data we are presenting covers a period in the EU that includes both the ‘epidemic’ and the ‘post‐epidemic’ period following the intervention with the restriction of MI use in cosmetic products, which may introduce a carry‐over effect. This contrasts with the United States, where MI use has not been as aggressively restricted. Despite this, the geographical differences provide noteworthy insight into the impact fullness of recent EU regulations [54, 55]. On the other hand, unfortunately, a similar notion has not been seen in the United States, where regulatory authorities have been less proactive [48].

### 4.4 Geographical Regions

Generally, geographical differences were observed with a tendency towards lower prevalences of PPTs to several allergens in Europe compared to the United States, Asia and the Middle East. The geographical variations are a multifaceted phenomenon influenced by multiple factors including environmental, cultural and regulatory factors. Comparing Europe to the United States, notable differences should be considered regarding tightened regulatory measures in Europe compared to the United States.

Furthermore, while the estimates presented in this study provide highly needed information about the prevalence of CA in children in different regions, most estimates rely on a few studies with a low number of children. Thus, the prevalence of CA in some allergens should be interpreted with caution. Despite this, variations in patch test reporting could be influenced by regional diagnostic practices and environmental exposures [56]. Moreover, the results presented in this study reveal a lack of epidemiological studies on the prevalence of CA in Asia and the Middle East. Although several population‐based epidemiological studies have been conducted in Europe [48, 57, 58], similar studies are highly needed for other regions. The observed discrepancies in the prevalence of CA between Western regions, in comparison to Asia and the Middle East, serve to reinforce the notion that the latter cannot rely on estimates derived from the Western world to inform local clinical practice. This underscores the necessity for further region‐specific research.

### 4.5 Atopic Dermatitis

The data did not allow for comparisons between children with and without AD, so all comparisons were made between all children and those with AD. We did find a higher proportion of PPTs to nickel among children with AD compared to all children. In children with AD, the compromised skin barrier may facilitate increased penetration of allergens like nickel, thus exacerbating allergic responses [59, 60]. ACD in people with AD is a complex interplay of immunological pathways. Although the traditional explanation considered suppression of T‐helper cell lineages [61, 62], newer explanations focus on inflammatory cytokines, namely interleukin‐4 [25], as well as innate lymphoid cells, which play a pivotal role in modulating immune responses and directing T‐cells to sites of inflammation and repair in AD [63, 64]. Furthermore, patients with AD and filaggrin mutations have been found to have higher rates of sensitisation to nickel [65].

Regarding fragrances, we found increased pooled prevalences of FM I (6.6% vs. 4.8%) and FM II (3.6% vs. 3.0%) in children with AD compared to all children. This is a slight difference compared to a recent systematic review in European paediatric dermatitis patients, revealing a prevalence of 4.1% and 2.2% for FM I and FM II, respectively [66]. CA to fragrances was also found to increase in persons with AD in a general population study, which was thought to be due to the increased exposure of persons with AD to moisturisers many of which would contain fragrance ingredients [67]. Importantly, concurrent CA and AD in children might delay the diagnosis due to similar clinical appearance and localisation. This highlights the importance of recognition of clinical aspects of concurrent ACD and AD, as this may prevent the delay of diagnosis [28, 68].

We found CA to carba mix to be more prevalent in children with AD and CA to thiuram mix to be more prevalent in all children. However, a recent study has indicated that there is no association between CA to thiuram mix and AD in adults [69]. Interestingly, children with AD had a significantly higher proportion of PPTs for cocamidopropyl betaine (5.5% vs. 9%, *p* = 0.0030) and propylene glycol (3.2% vs. 6.6%, *p* = 0.0006). These substances have been classified as irritants and weak allergens [70, 71]. Furthermore, it is presumed that the vast majority of PPTs to cocamidopropyl betaine are false positive, with allergic reactions being rare [70].

### 4.6 Study Strengths and Limitations

The strengths of this study include the comprehensive literature search with more than 12 000 articles screened based on title and abstract, thus lowering the risk of missing reports. Further, while only 17 studies were included, the studies had relatively large populations and comprised a total of 11 593 children. This allowed for an estimate of the true prevalence of allergens responsible for CA in children. However, when interpreting the results from this study, some limitations need to be considered. For most allergens, high between‐study heterogeneity was found, most likely due to geographical and study‐related factors, which could affect the prevalence estimates. On the other hand, most studies used the same concentration and vehicle for the respective allergens, which facilitates greater comparability across studies. Although some allergens were reported in a limited number of studies, the sample size was typically large. Additionally, allergens selected for testing in children at many centres are often based on prevalent allergens observed in adult populations from the same geographical regions. As such, the allergens tested may not fully reflect the unique exposures of paediatric patients, and the importance of case reports, such as those involving rare allergens like carmine, should not be overlooked. While acknowledging these limitations, we offer a comprehensive estimate of the current prevalence of PPTs in allergens responsible for CA in children.

## 5 Conclusions

CA is frequent in children across different geographical regions, though there are significant differences in the pooled prevalence of high‐prevalence allergens such as nickel, MCI/MI, MI and fragrances. These variations solely depend on regulatory interventions to highlight their importance. The observed waxing and waning of allergen prevalence underscores the importance of consistent regulatory oversight to minimise the risk of emerging sensitivities and protect public health. Based on the included studies, the children were routinely patch tested with a total of 52 allergens, many of these (*n* = 35) with a PPTs in more than 1% of the cases and even a few are known to cause irritation rather than CA, for example, cocamide. With this in mind, we emphasise the need for standardisation and patch test recommendations in children. Hopefully, the results presented herein may contribute to the development of a paediatric baseline series, thus enhancing paediatric patch testing.

## Author Contributions

**Daniel Isufi:** writing – original draft, writing – review and editing, methodology, validation, data curation, conceptualization, investigation, visualization, project administration, resources. **Mikkel Bak Jensen:** conceptualization, investigation, writing – review and editing, visualization, software, formal analysis, data curation, supervision, resources. **Christoffer Kursawe Larsen:** writing – review and editing, validation, investigation, supervision. **Farzad Alinaghi:** supervision, visualization, writing – review and editing, validation, methodology, software. **Jakob Ferløv Baselius Schwensen:** writing – review and editing, supervision, validation. **Jeanne D. Johansen:** writing – review and editing, conceptualization, investigation, supervision, validation, visualization.

## Conflicts of Interest

The authors declare no conflicts of interest. [Correction added on 25 March 2025, after first online publication: Conflict of interest was inadvertently removed and has been reinstated in this version.]

## Supporting information


**Data S1.** Supporting Information.

## Data Availability

The data that support the findings of this study are available through the corresponding author.
