# Supplementary material for: Allergens Responsible for Contact Allergy in Children From 2010 to 2024: A Systematic Review and Meta‐Analysis
Source: Contact Dermatitis. 2025 Jan 19;92(5):327–43. doi: 10.1111/cod.14753 (PMC11965549; doi:10.1111/cod.14753)
Supplement: Supplementary file 1 — Data S1. Supporting Information. [file COD-92-327-s001.docx]

**Supplementary table 1**. Appraisal tool for cross sectional studies (AXIS).

|  | **Introduction** | **Methods** | | | | | | | | | | **Results** | | | | | **Discussion** | | **Other** | | |
| --- | --- | --- | --- | --- | --- | --- | --- | --- | --- | --- | --- | --- | --- | --- | --- | --- | --- | --- | --- | --- | --- |
| **Reference** | **Q1** | **Q2** | **Q3** | **Q4** | **Q5** | **Q6** | **Q7** | **Q8** | **Q9** | **Q10** | **Q11** | **Q12** | **Q13** | **Q14** | **Q15** | **Q16** | **Q17** | **Q18** | **Q19** | **Q20** |  |
| Bonamonte (2022) (1) | **1** | **1** | **1** | **1** | **1** | **1** | **1** | **1** | **1** | **1** | **1** | **1** | **1** | **1** | **1** | **1** | **1** | **2** | **1** | **1** |  |
| Fortina (2016) (2) | **1** | **1** | **1** | **1** | **1** | **1** | **1** | **1** | **1** | **1** | **1** | **1** | **1** | **1** | **1** | **1** | **1** | **2** | **3** | **3** |  |
| Siemund (2022) (3) | **1** | **1** | **1** | **1** | **1** | **1** | **1** | **1** | **1** | **1** | **1** | **1** | **1** | **1** | **1** | **1** | **1** | **2** | **1** | **1** |  |
| Lagrelius (2016) (4) | **1** | **1** | **1** | **1** | **1** | **1** | **1** | **1** | **1** | **1** | **1** | **1** | **1** | **1** | **1** | **1** | **1** | **1** | **1** | **1** |  |
| Machovcova (2012) (5) | **1** | **1** | **1** | **1** | **1** | **1** | **2** | **1** | **1** | **2** | **1** | **1** | **1** | **1** | **1** | **2** | **1** | **2** | **3** | **3** |  |
| Kakşi (2022) (6) | **1** | **1** | **1** | **1** | **1** | **1** | **1** | **1** | **1** | **1** | **1** | **1** | **1** | **3** | **1** | **1** | **1** | **1** | **1** | **1** |  |
| Young (2023) (7) | **1** | **1** | **1** | **1** | **1** | **1** | **1** | **1** | **1** | **2** | **3** | **1** | **1** | **1** | **1** | **3** | **1** | **3** | **1** | **3** |  |
| Simonsen (2018) (8) | **1** | **1** | **1** | **1** | **1** | **1** | **1** | **1** | **1** | **1** | **1** | **1** | **1** | **1** | **1** | **1** | **1** | **1** | **1** | **1** |  |
| Johnson (2023) (9) | **1** | **1** | **1** | **1** | **1** | **1** | **1** | **1** | **1** | **1** | **1** | **1** | **1** | **1** | **1** | **1** | **1** | **1** | **1** | **1** |  |
| Boonchai (2021) (10) | **1** | **1** | **1** | **1** | **1** | **1** | **1** | **1** | **1** | **1** | **1** | **1** | **1** | **1** | **1** | **1** | **1** | **1** | **1** | **1** |  |
| Handa (2024) (11) | **1** | **1** | **1** | **1** | **1** | **1** | **1** | **1** | **1** | **1** | **1** | **1** | **1** | **1** | **1** | **1** | **1** | **1** | **1** | **1** |  |
| Christiansen (2016) (12) | **1** | **1** | **1** | **1** | **1** | **1** | **1** | **1** | **1** | **1** | **1** | **1** | **1** | **1** | **1** | **1** | **1** | **1** | **1** | **1** |  |
| Jacob (2017) (13) | **1** | **1** | **1** | **1** | **1** | **1** | **1** | **1** | **1** | **1** | **1** | **1** | **1** | **1** | **1** | **1** | **1** | **1** | **1** | **1** |  |
| Andre (2024) (14) | **1** | **1** | **1** | **1** | **1** | **1** | **1** | **1** | **1** | **1** | **1** | **1** | **1** | **1** | **1** | **1** | **1** | **1** | **1** | **1** |  |
| Slodownik (2023) (15) | **1** | **1** | **1** | **1** | **1** | **1** | **1** | **1** | **1** | **1** | **1** | **1** | **1** | **1** | **1** | **1** | **1** | **1** | **1** | **1** |  |
| Barwari (2023) (16) | **1** | **1** | **1** | **1** | **1** | **1** | **1** | **1** | **1** | **1** | **1** | **1** | **1** | **1** | **1** | **1** | **1** | **1** | **1** | **1** |  |
| Noë (2022) (17) | **1** | **1** | **1** | **1** | **1** | **1** | **1** | **1** | **1** | **1** | **1** | **1** | **1** | **1** | **1** | **1** | **1** | **1** | **1** | **1** |  |
| **Abbreviations:** Q, question; 1, yes; 2, no; 3, don’t know/not applicable. | | | | | | | | | | | | | | | | | | | | |  |

**Supplementary figure 1**. Funnel plot of studies reporting nickel sulphate in all children


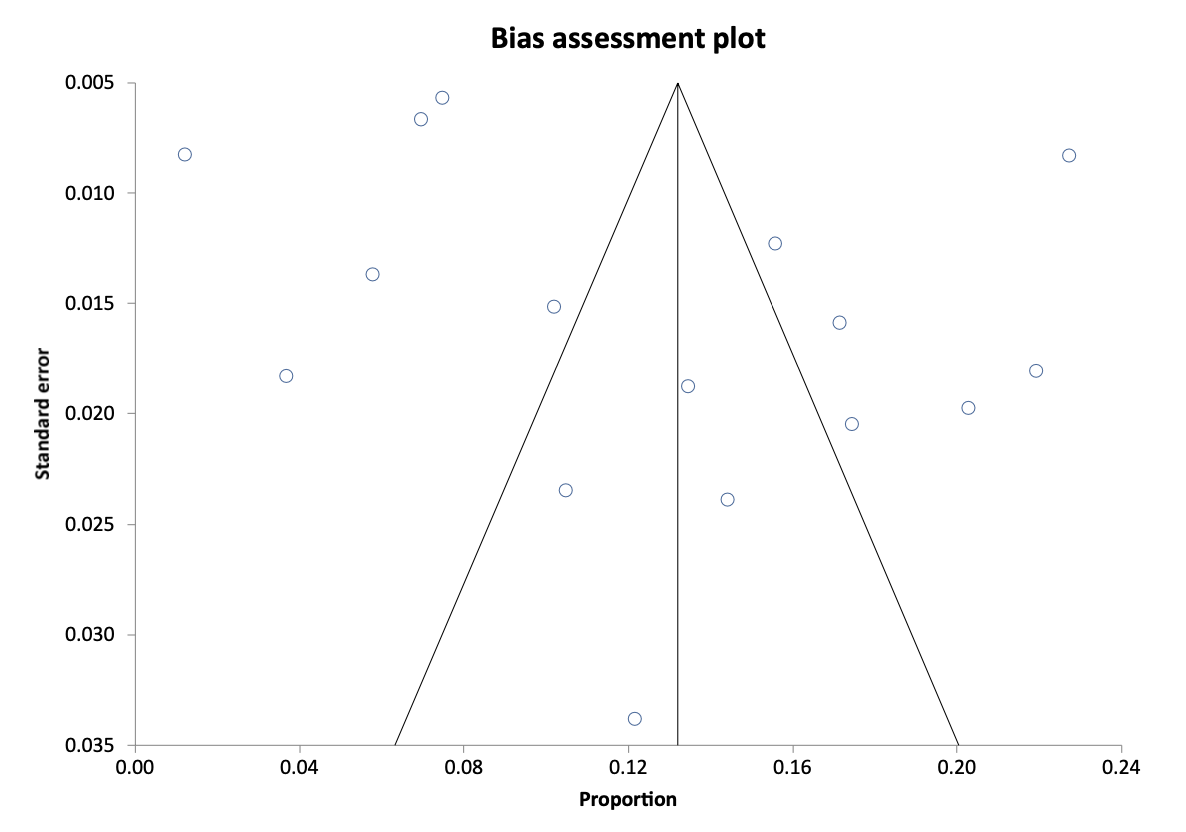


**Supplementary figure 2**. Funnel plot of studies reporting nickel sulphate in children with atopic dermatitis


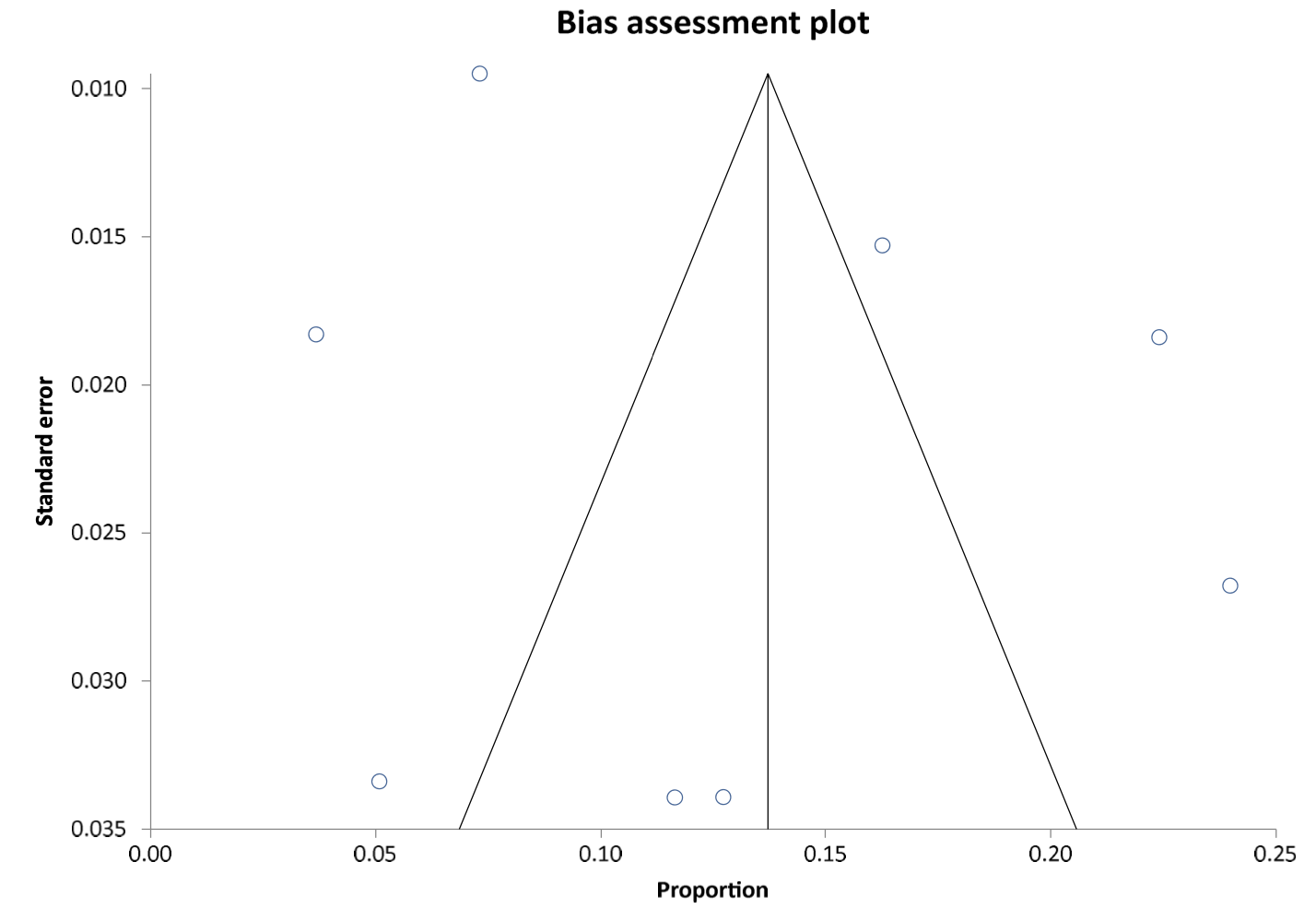


**References**

1. Bonamonte D, Hansel K, Romita P, Fortina AB, Girolomoni G, Fabbrocini G, et al. Contact allergy in children with and without atopic dermatitis: An Italian multicentre study. Contact Dermatitis [Internet]. 2022 Sep 1 [cited 2024 May 31];87(3):265. Available from: /pmc/articles/PMC9541036/

2. Belloni Fortina A, Fontana E, Peserico A. Contact Sensitization in Children: A Retrospective Study of 2,614 Children from a Single Center. Pediatr Dermatol [Internet]. 2016 Jul 1 [cited 2024 May 31];33(4):399–404. Available from: https://onlinelibrary-wiley-com.ep.fjernadgang.kb.dk/doi/full/10.1111/pde.12873

3. Siemund I, Dahlin J, Hindsén M, Zimerson E, Antelmi A, Hamnerius N, et al. Contact Allergy to Two Aluminum Salts in Consecutively Patch-Tested Dermatitis Patients. Dermatitis [Internet]. 2022 [cited 2024 Jul 2];33(1):31–5. Available from: https://pubmed.ncbi.nlm.nih.gov/34570735/

4. Lagrelius M, Wahlgren CF, Matura M, Kull I, Lidén C. High prevalence of contact allergy in adolescence: results from the population-based BAMSE birth cohort. Contact Dermatitis [Internet]. 2016 Jan 1 [cited 2024 Jul 2];74(1):44–51. Available from: https://onlinelibrary-wiley-com.ep.fjernadgang.kb.dk/doi/full/10.1111/cod.12492

5. Machovcová A, Machovcova A. The Frequency of Contact Allergy in Children and Adolescents in the Czech Republic. ACTA DERMATOVENEROLOGICA CROATICA Acta Dermatovenerol Croat [Internet]. 2012 [cited 2024 Jul 2];20(2):75–9. Available from: https://www.researchgate.net/publication/228060245

6. Kakşi SA, Kahraman FC, Akdeniz N, Özen T. Results of the patch tests with European baseline series in children: Five years of experience from a single center in Turkey and a review of the literature. J Cosmet Dermatol [Internet]. 2023 Mar 1 [cited 2024 Jul 3];22(3):1071–6. Available from: https://onlinelibrary-wiley-com.ep.fjernadgang.kb.dk/doi/full/10.1111/jocd.15531

7. Young K, Collis RW, Sheinbein D, Shope C, Suresh T, Tam I, et al. Pediatric Allergic Contact Dermatitis Registry patch testing results from 2016 to 2022: A retrospective study of age-related differences. J Am Acad Dermatol. 2023 May 1;88(5):1218–20.

8. Simonsen AB, Foss-Skiftesvik MH, Thyssen JP, Deleuran M, Mortz CG, Zachariae C, et al. Contact allergy in Danish children: Current trends. Contact Dermatitis [Internet]. 2018 Nov 1 [cited 2024 Jul 3];79(5):295–302. Available from: https://onlinelibrary-wiley-com.ep.fjernadgang.kb.dk/doi/full/10.1111/cod.13079

9. Johnson H, Aquino MR, Snyder A, Collis RW, Franca K, Goldenberg A, et al. Prevalence of allergic contact dermatitis in children with and without atopic dermatitis: A multicenter retrospective case-control study. J Am Acad Dermatol. 2023 Nov 1;89(5):1007–14.

10. Boonchai W, Chaiyabutr C, Charoenpipatsin N, Sukakul T. Pediatric contact allergy: A comparative study with adults. Contact Dermatitis [Internet]. 2021 Jan 1 [cited 2024 Jul 3];84(1):34–40. Available from: https://onlinelibrary-wiley-com.ep.fjernadgang.kb.dk/doi/full/10.1111/cod.13672

11. Handa S, Bhattacharjee R, Thakur V, De D, Mahajan R. Contact hypersensitivity to Indian standard patch test series correlates with disease severity among children with atopic dermatitis. Indian J Dermatol Venereol Leprol. 2024;90:46–51.

12. Christiansen ES, Andersen KE, Bindslev-Jensen C, Halken S, Kjaer HF, Eller E, et al. Low patch test reactivity to nickel in unselected adolescents tested repeatedly with nickel in infancy. Pediatric Allergy and Immunology [Internet]. 2016 Sep 1 [cited 2024 Jul 4];27(6):636–9. Available from: https://onlinelibrary-wiley-com.ep.fjernadgang.kb.dk/doi/full/10.1111/pai.12578

13. Jacob SE, McGowan M, Silverberg NB, Pelletier JL, Fonacier L, Mousdicas N, et al. Pediatric Contact Dermatitis Registry Data on Contact Allergy in Children With Atopic Dermatitis. JAMA Dermatol [Internet]. 2017 Aug 1 [cited 2024 Jul 4];153(8):765–70. Available from: https://jamanetwork-com.ep.fjernadgang.kb.dk/journals/jamadermatology/fullarticle/2604252

14. Andre N, Usher A, Ofri M, Kilimnik V, Horev A. Examining the benefits of extended patch test series in children: a comprehensive analysis. Int J Dermatol [Internet]. 2024 [cited 2024 Jul 4]; Available from: https://onlinelibrary-wiley-com.ep.fjernadgang.kb.dk/doi/full/10.1111/ijd.17103

15. Slodownik D, Bar J, Solomon M, Lavy Y, Baum S, Galed OM, et al. Pediatric Contact Dermatitis: A 10-Year Multicenter Retrospective Study. 2023 [cited 2024 Jul 4]; Available from: www.liebertpub.com

16. Barwari L, Rustemeyer T, Franken SM, Ipenburg NA. Patch test results in a Dutch paediatric population with suspected contact allergy: A retrospective cohort study. Contact Dermatitis [Internet]. 2023 Feb 1 [cited 2024 Jul 5];88(2):120–8. Available from: https://onlinelibrary-wiley-com.ep.fjernadgang.kb.dk/doi/full/10.1111/cod.14231

17. Noë E, Huygens S, Morren MA, Garmyn M, Goossens A, Gilissen L. Contact allergy in a paediatric population observed in a tertiary referral centre in Belgium. Contact Dermatitis [Internet]. 2022 Jan 1 [cited 2024 Jul 5];86(1):3–8. Available from: https://onlinelibrary-wiley-com.ep.fjernadgang.kb.dk/doi/full/10.1111/cod.13975
